# Impact of the common *MTHFR* 677C→T polymorphism on blood pressure in adulthood and role of riboflavin in modifying the genetic risk of hypertension: evidence from the JINGO project

**DOI:** 10.1186/s12916-020-01780-x

**Published:** 2020-11-11

**Authors:** Mary Ward, Catherine F. Hughes, J. J. Strain, Rosie Reilly, Conal Cunningham, Anne M. Molloy, Geraldine Horigan, Miriam Casey, Kevin McCarroll, Maurice O’Kane, Michael J. Gibney, Albert Flynn, Janette Walton, Breige A. McNulty, Adrian McCann, Laura Kirwan, John M. Scott, Helene McNulty

**Affiliations:** 1 The Nutrition Innovation Centre for Food and Health (NICHE), School of Biomedical Sciences, Ulster University, Coleraine, Northern Ireland, UK; 2 The Department of Gerontology, St James’s Hospital, Dublin, Ireland; 3 School of Medicine and School of Biochemistry and Immunology, Trinity College Dublin, Dublin, Ireland; 4 Clinical Chemistry Laboratory, Western Health and Social Care Trust, Altnagelvin Hospital, Londonderry, Northern Ireland, UK; 5 School of Agriculture and Food Science, University College Dublin, Dublin, Ireland; 6 School of Food and Nutritional Sciences, University College Cork, Cork, Ireland

**Keywords:** Hypertension, Blood pressure, Folate polymorphism, MTHFR, Riboflavin, Personalised treatment, Prevention

## Abstract

**Background:**

Genome-wide and clinical studies have linked the 677C→T polymorphism in the gene encoding methylenetetrahydrofolate reductase (MTHFR) with hypertension, whilst limited evidence shows that intervention with riboflavin (i.e. the MTHFR co-factor) can lower blood pressure (BP) in hypertensive patients with the variant *MTHFR* 677TT genotype. We investigated the impact of this common polymorphism on BP throughout adulthood and hypothesised that riboflavin status would modulate the genetic risk of hypertension.

**Methods:**

Observational data on 6076 adults of 18–102 years were drawn from the Joint Irish Nutrigenomics Organisation project, comprising the Trinity-Ulster Department of Agriculture (TUDA; volunteer sample) and the National Adult Nutrition Survey (NANS; population-based sample) cohorts. Participants were recruited from the Republic of Ireland and Northern Ireland (UK) in 2008–2012 using standardised methods.

**Results:**

The variant *MTHFR* 677TT genotype was identified in 12% of adults. From 18 to 70 years, this genotype was associated with an increased risk of hypertension (i.e. systolic BP ≥ 140 and/or a diastolic BP ≥ 90 mmHg): odds ratio (OR) 1.42, 95% confidence interval (CI) 1.07 to 1.90; *P* = 0.016, after adjustment for antihypertensive drug use and other significant factors, namely, age, male sex, BMI, alcohol and total cholesterol. Low or deficient biomarker status of riboflavin (observed in 30.2% and 30.0% of participants, respectively) exacerbated the genetic risk of hypertension, with a 3-fold increased risk for the TT genotype in combination with deficient riboflavin status (OR 3.00, 95% CI, 1.34–6.68; *P* = 0.007) relative to the CC genotype combined with normal riboflavin status. Up to 65 years, we observed poorer BP control rates on antihypertensive treatment in participants with the TT genotype (30%) compared to those without this variant, CT (37%) and CC (45%) genotypes (*P* < 0.027).

**Conclusions:**

The *MTHFR* 677TT genotype is associated with higher BP independently of homocysteine and predisposes adults to an increased risk of hypertension and poorer BP control with antihypertensive treatment, whilst better riboflavin status is associated with a reduced genetic risk. Riboflavin intervention may thus offer a personalised approach to prevent the onset of hypertension in adults with the TT genotype; however, this requires confirmation in a randomised trial in non-hypertensive adults.

## Background

Hypertension is the leading risk factor contributing to all-cause death in every region in the world, estimated to affect 1.13 billion people globally and account for over 9 million deaths annually, predominantly from cardiovascular disease (CVD) [1–3]. The relationship of blood pressure (BP) with disease is age-specific and most pronounced in adults 40–69 years, where the risk of CVD is estimated to double for each 20 mmHg rise in systolic BP [4]. Recent reports have highlighted the importance of targeting lifestyle and treatment strategies at the individual level in order to improve cardiovascular health [1, 5], and genome-wide association studies (GWAS) have identified specific genes linked with BP which could lead to personalised treatments for hypertension based on genetic characteristics. The earliest of such studies tested 2.5 million single nucleotide polymorphisms (SNPs) and identified eight genetic loci associated with BP, including a region near the gene encoding the folate-metabolising enzyme methylenetetrahydrofolate reductase MTHFR [6], findings confirmed by subsequent GWAS [7].

Of greater relevance to health, clinical studies have linked this gene with BP, with meta-analyses of case-control studies showing that the 677C→T polymorphism in *MTHFR* is associated with an increased risk of hypertension by 36–87% [8–10]. Previously, the role of this polymorphism in CVD has been studied extensively in relation to the well-recognised phenotype, elevated homocysteine, whilst the relationship with BP is relatively under-investigated. The variant *MTHFR* 677TT genotype, which affects 10% of adults worldwide [11], is however reported to increase the risk of CVD (especially stroke) by up to 40%, albeit with a large geographical variation in the extent of excess risk, consistent with a gene-environment interaction [12–14]. In this regard, only folate was previously considered, but emerging evidence suggests that riboflavin—required in the form flavin adenine dinucleotide (FAD) as a cofactor for MTHFR—may be a key modifying factor linking this polymorphism with CVD via a novel and genotype-specific effect on BP [14]. In three small randomised controlled trials, we previously demonstrated lowering of systolic BP by 6 to 13 mmHg in response to riboflavin when targeted at hypertensive patients with the variant *MTHFR* 677TT genotype [15–17].

No previous study has investigated the contribution of the *MTHFR* 677C→T polymorphism to BP within generally healthy adults or identified a potential prevention strategy to reduce the onset of hypertension in those genetically at-risk. The aim of this study was therefore to examine the impact of this polymorphism on BP throughout adulthood and to assess the role of riboflavin in modulating the genetic risk of hypertension. We hypothesised that this polymorphism is associated with high BP independently of its association with homocysteine and that riboflavin status would modulate the genetic risk of hypertension.

## Methods

### Design and participants

Data for this investigation were drawn from two cohorts, the Trinity-Ulster Department of Agriculture (TUDA) cohort study and the National Adult Nutrition Survey (NANS) of Ireland, both forming part of an All-Ireland initiative under the Joint Irish Nutrigenomics Organisation (JINGO) project (http://www.ucd.ie/jingo/; accessed May 2020). The TUDA study (ClinicalTrials.gov Identifier: NCT02664584) comprises a cross-sectional cohort of 5186 older adults (≥ 60 years), with the primary aim of investigating nutritional factors and gene-nutrient interactions in the development of chronic diseases of ageing. Eligible participants were community-dwelling, non-institutionalised adults, born on the island of Ireland. Participants were recruited using standardised protocols during the period of 2008 to 2012, either from GP practices in the Northern and Western Trusts in Northern Ireland (UK) or from hospital outpatient clinics at the Department of Medicine for the Elderly at St. James’s Hospital Dublin in the Republic of Ireland, as previously detailed [18]. Over a similar period (2008 to 2010), detailed dietary, biomarker, health and lifestyle data were collected for the NANS cohort, a nationally representative sample of Irish adults. Eligible participants were healthy adults aged 18–102 years, not pregnant or breast-feeding. Full sampling and methodological details for NANS 2008–2010 have been described elsewhere [19]. Approval for both studies was granted from the relevant ethics committees in the UK and the Republic of Ireland, and all participants provided written informed consent at the time of recruitment.

### Study measurements

For both the TUDA and NANS cohorts, relevant health and lifestyle information was obtained in face-to-face interviews conducted by trained researchers. Detailed information concerning medication and vitamin supplement usage was collected. Confirmation of medication details was obtained by referring to the participant’s prescription; where this was unavailable during the interview, the details were collected from the participant via telephone shortly after the appointment. Blood samples collected at the time of the appointment were analysed for routine laboratory measurements in the participating local laboratories, whereas B vitamin status biomarkers were analysed centrally in specialist research laboratories at Ulster University or Trinity College Dublin using standardised procedures [16]. Of particular relevance, the analysis included the riboflavin biomarker, erythrocyte glutathione reductase activation coefficient (EGRac), widely accepted as the gold-standard measure of riboflavin status. This coefficient provides a measure of glutathione reductase enzyme saturation with its riboflavin-derived cofactor and is thus a functional biomarker of riboflavin status; a low EGRac value is considered to be normal, whilst higher values are indicative of suboptimal riboflavin status. DNA samples were analysed for several SNPs, including *MTHFR* 677C→T (rs1801133), by LGC Genomics (Hoddesdon, UK).

Trained researchers measured BP using standard operating procedures and clinical guidelines, using an A&D UA-787 digital blood pressure monitor (Cardiac Services, Belfast, UK) or OMRON M6 (Milton Keynes, UK), for TUDA and NANS cohorts, respectively, with the participant in the supine position following a 5-min rest. In accordance with clinical guidelines [20], two BP measurements were taken from the reference arm, with a 5–10-min interval between each measurement to generate a mean BP value. In the case of a > 5-mmHg difference, a third BP measurement was taken after 10–15 min and the mean of the two BP measurements in closest agreement was used.

### Study outcomes

The primary outcomes were systolic and diastolic BP, and occurrence of hypertension, by *MTHFR* genotype and *MTHFR*-riboflavin interaction. In accordance with British and European guidelines, hypertension was defined when a participant’s systolic BP was ≥ 140 mmHg and/or their diastolic BP was ≥ 90 mmHg; as per clinical guidance, these BP categories applied to all adults (> 18 years) [1, 20]. An additional study outcome was BP control on antihypertensive treatment by *MTHFR* genotype. Treatment was defined as taking medication to lower BP, as verified by the researcher against prescription details during or following the interview. Treated and controlled was defined as taking medication to lower BP and a recorded systolic BP of < 140 and/or diastolic BP < 90 mmHg.

### Statistical analysis

**Fig. 1:**
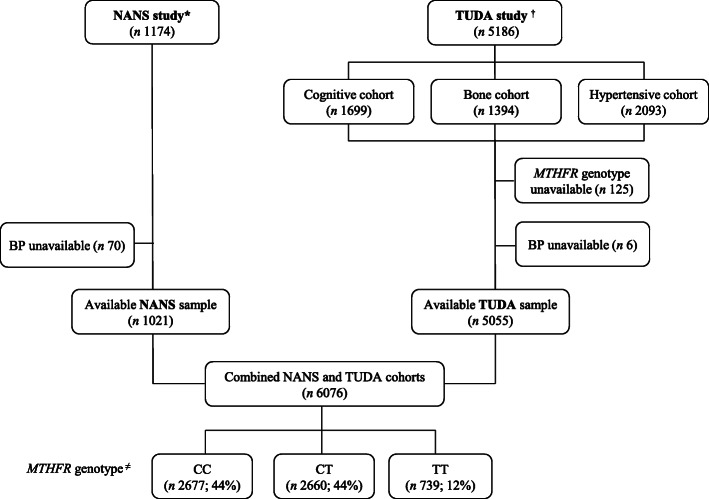
Identification of study participants from two cohorts under the Joint Irish Nutrigenomics (JINGO) Initiative. *National Adult Nutrition Survey of Ireland. ^†^Trinity-Ulster and Department of Agriculture cohort study. ^≠^CC (wild type), CT (heterozygous), TT (homozygous), genotypes for the 677C→T polymorphism in *MTHFR*

Analysis was limited to participants with available *MTHFR* genotype and valid BP (Fig. 1). Before statistical analysis, tests for normality were performed and variables were log-transformed as appropriate. Participant characteristics were examined by *MTHFR* genotype and differences between groups were analysed using one-way between-groups analysis of variance (ANOVA) for continuous variables and *χ*^2^ tests for categorical parameters. To account for multiple testing, the null hypothesis was rejected for *P* < 0.05 after post hoc Bonferroni correction at a family level. Logistic regression analysis was used to predict hypertension (as the categorical dependent variable) using relevant independent variables and examined the association of *MTHFR* genotype with the risk of hypertension after independently adjusting for established risk factors, including antihypertensive drug use (as a binary yes/no covariate adjustment). Multinomial regression was performed to enable the effect of the interaction between *MTHFR* genotype and biomarker status of riboflavin (i.e. deficient versus low versus normal) on the risk of hypertension to be assessed; odds ratios were calculated using *MTHFR* 677CC genotype combined with normal riboflavin status as the reference category. Statistical analysis was performed using the Statistical Package for Social Sciences (SPSS, version 21, SPSS UK Ltd., Chertsey Road, Surrey, UK).

## Results

### Study participants

From an original dataset of 6360 participants (i.e. combined TUDA [*n* = 5186] and NANS [*n* = 1174] cohorts), complete data for the current analysis were available for a total of 6076 participants (Fig. 1). Homozygosity for the *MTHFR* 677C→T polymorphism (TT genotype) was identified in 12% of the overall study sample (12.1% and 12.3% for TUDA and NANS cohorts, respectively; Additional file 1: Table S1 showing characteristics separately presented for TUDA and NANS cohorts). There were no significant differences in general participant characteristics among *MTHFR* genotype groups (Table 1). The expected phenotype was however evident in B vitamin biomarkers, with significantly higher plasma homocysteine and lower red blood cell folate concentrations in the TT compared to CC or CT genotypes. General participant characteristics split by study sub-cohorts (i.e. TUDA and NANS cohorts) are provided as Supplementary material (Additional file 1: Table S1).

**Table 1:** General participant characteristics by *MTHFR* genotype

	*MTHFR* genotype^a^			
	CC (*n* = 2677)	CT (*n* = 2660)	TT (*n* = 739)	*P* value^b^
*MTHFR* genotype, *n* (%)	2677 (44)	2660 (44)	739 (12)	
Age, years	68.9 (15.1)	69.0 (15.5)	68.6 (15.6)	0.806
Sex, male	943 (35%)	961 (36%)	256 (35%)	0.678
Waist, cm	94.5 (13.9)	94.5 (14.1)	94.7 (14.7)	0.982
Height, cm	162.6 (10.2)	162.9 (10.1)	162.6 (10.0)	0.619
Weight, kg	73.7 (16.5)	73.7 (16.8)	74.2 (17.3)	0.853
Body mass index, kg/m^2^	27.8 (5.2)	27.7 (5.4)	27.9 (5.2)	0.458
Current smokers, *n* (%)	359 (13%)	355 (13%)	89 (12%)	0.530
Alcohol Intake, units/week	8.6 (12.2)	8.8 (12.7)	8.0 (11.3)	0.402
Serum triglycerides, mmol/L	1.51 (0.84)	1.56 (0.88)	1.55 (0.78)	0.087
Serum total cholesterol, mmol/L	4.68 (1.03)	4.68 (1.06)	4.73 (1.05)	0.383
Serum HDL, mmol/L	1.51 (0.49)	1.48 (0.45)	1.49 (0.47)	0.439
Calculated LDL, mmol/L	2.50 (0.88)	2.50 (0.89)	2.54 (0.88)	0.472
Serum creatinine, μmol/L	86.3 (27.4)	86.0 (26.4)	85.9 (27.2)	0.928
B-vitamin biomarkers				
Red blood cell folate, nmol/L	1095 (579)^a^	1088 (626)^a^	971 (563)^b^	< 0.001
Serum vitamin B12, pmol/L	295 (155)	295 (238)	296 (238)	0.194
Riboflavin status, EGRac^c^	1.35 (0.21)	1.35 (0.21)	1.34 (0.21)	0.769
Plasma homocysteine, μmol/L	14.2 (5.4)^a^	14.3 (5.4)^a^	15.7 (6.8)^b^	< 0.001

Data are expressed as mean (standard deviation) or *n* (%).

^a^CC (wild type), CT (heterozygous), TT (homozygous variant), genotypes for the *MTHFR* 677C→T polymorphism

^b^*P* value from one-way ANOVA comparing genotype groups, following log-transformation of data for normalisation purposes, as appropriate. Different superscript letters (i.e. a, b) within a row indicate significant differences by Bonferroni post hoc test, whilst the same letter (i.e. a, a) indicates no significant differences. Level of significance (*P* < 0.003) adjusted for Bonferroni correction (*n* = 16). Categorical variables are assessed using chi-square analysis

^c^Biomarker status of riboflavin determined by the functional assay, erythrocyte glutathione reductase activation coefficient (EGRac); higher EGRac values indicate lower riboflavin status

### Impact of *MTHFR* genotype on blood pressure and risk of hypertension

**Fig. 2:**
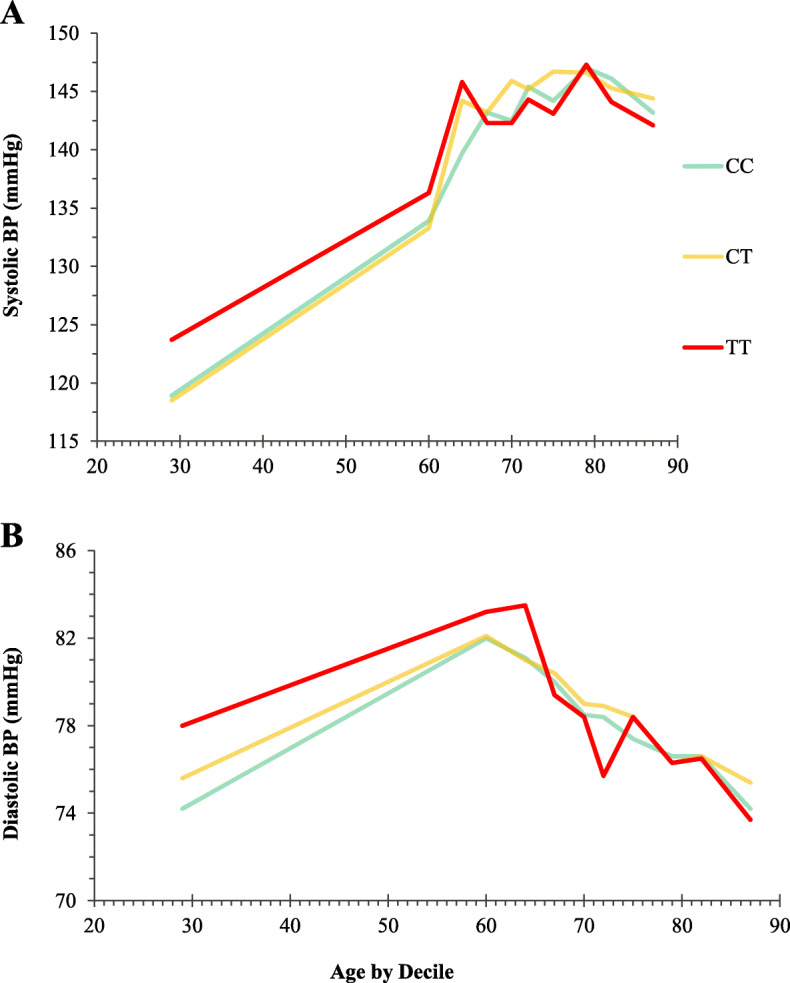
Systolic and diastolic blood pressure in adults 18–90 years by *MTHFR* genotype (*n =* 6070). Data grouped by deciles of age from the youngest 10%, to the oldest 10%, of study participants. Each line illustrates median systolic or diastolic blood pressure for adults by age: CC (green line), CT (amber line) and TT (red line) genotypes for the *MTHFR* 677C→T polymorphism

Irrespective of *MTHFR* genotype, systolic BP showed an increase with age up to approximately 80 years, whereas diastolic BP increased until about age 60 years and then declined (Fig. 2). Examination of BP by *MTHFR* genotype, however, showed higher BP in the TT genotype group up to approximately 65 years compared to adults of the same age with CC or CT genotypes, with systolic and diastolic BP in the TT genotype observed to be typical of an adult several years older without this genetic variant. From about 65 years onwards, however, the BP phenotype associated with this polymorphism was less evident.

Among adults 18–70 years, logistic regression analysis showed that the *MTHFR* 677TT genotype was associated with an increased risk of hypertension: odds ratio (OR) 1.42, 95% confidence interval (CI) 1.07 to 1.90, after adjustment for antihypertensive drug use (as a binary covariate) and other significant covariates, namely, age, male sex, BMI, alcohol, total cholesterol and study cohort (Table 2), whereas homocysteine was not independently associated with the risk of hypertension (apart from in treated adults). The OR for risk of hypertension associated with the TT genotype remained similar whether the logistic regression analysis was performed in all participants up to 70 years, or split into those treated or not treated with antihypertensive drugs, albeit the relationship failed to reach statistical significance within either treated or untreated categories (owing to the loss of statistical power as a result of a 50% reduction in the sample size when split and considering that the variant TT genotype is represented by just 12% of the overall cohort). In contrast, when this analysis was conducted in the total sample (i.e. adults up to 90 years), *MTHFR* genotype was not significantly associated with hypertension, whilst all other determinants of hypertension were similar to those found in adults up to 70 years (not shown).

**Table 2:** Factors associated with risk of hypertension in adults 18–70 years

	All (*n* = 2566)			On antihypertensive drugs (*n* = 1255)			Not on antihypertensive drugs (*n* = 1311)		
	OR	95% CI	*P*^b^	OR	95% CI	*P*^b^	OR	95% CI	*P*^a^
Age, years	1.04	1.03–1.05	< 0.001	1.01	0.98–1.04	0.568	1.05	1.03–1.06	< 0.001
Sex, male	1.86	1.50–2.32	< 0.001	1.69	1.28–2.25	0.001	1.77	1.23–2.56	0.002
BMI, kg/m^2^	1.06	1.04–1.08	< 0.001	1.03	1.01–1.05	0.009	1.11	1.07–1.15	< 0.001
Alcohol intake, units per week	1.01	1.00–1.02	0.005	1.01	1.00–1.02	0.325	1.03	1.01–1.04	< 0.001
Antihypertensive medication use^b^	2.01	1.60–2.52	< 0.001						
Serum creatinine, μmol/L	1.00	0.99–1.00	0.307	1.00	0.99–1.00	0.189	1.01	0.99–1.02	0.340
Total cholesterol, mmol/L	1.26	1.15–1.38	< 0.001	1.25	1.11–1.41	< 0.001	1.23	1.07–1.41	0.004
Smoking									
Past	0.98	0.80–1.19	0.826	0.97	0.75–1.26	0.834	1.02	0.74–1.41	0.888
Current	1.03	0.79–1.33	0.845	0.92	0.64–1.31	0.630	1.10	0.75–1.62	0.618
Study cohort^c^	1.79	1.29–2.48	< 0.001	2.40	1.40–4.11	< 0.001	2.09	1.31–3.32	0.002
Plasma homocysteine, μmol/L	1.00	0.98–1.02	0.958	0.98	0.96–1.00	0.074	1.06	1.02–1.10	0.002
*MTHFR* genotype^d^									
CT	1.18	0.98–1.43	0.082	1.35	1.05–1.73	0.018	0.98	0.73–1.32	0.889
TT	1.42	1.07–1.90	0.016	1.40	0.95–2.06	0.093	1.37	0.88–2.11	0.161

*CI* confidence interval, *OR* odds ratio

^a^Data analysed by logistic regression to predict hypertension as the categorical dependent variable; hypertension defined as systolic BP of ≥ 140 and/or a diastolic BP of ≥ 90 mmHg [1]

^b^As a binary (yes/no) covariate

^c^Comparing TUDA cohort with NANS cohort (reference category). See supplementary Table S1 for participant characteristics presented separately for each study cohort

^d^CT (heterozygous) and TT (homozygous variant) genotypes for the *MTHFR* 677C→T polymorphism; reference category is the CC genotype

Likewise, no significant effect of *MTHFR* genotype on BP was observed when the total cohort was analysed, but among adults, 18 to 70 years, those with the TT genotype had significantly higher systolic and diastolic BP after adjustment for relevant covariates including antihypertensive drug use (Table 3). Among participants up to 70 years, 49% (*n* = 1255) were being treated with one or more antihypertensive drugs. Details of antihypertensive drug use and drug combinations among treated participants are shown in Table 4. Almost 60% of treated participants were treated with two or more medications (57%, 57% and 59% for CC, CT and TT genotypes). For BP results among participants being treated/not treated with antihypertensive drugs by MTHFR genotype, see Additional file 1: Table S2.

**Table 3:** Blood pressure and rates of hypertension in adulthood by *MTHFR* genotype

	*MTHFR* genotype			
	CC	CT	TT	*P* value^b^
Total cohort (up to 90 years)	*n* = 2635	*n* = 2606	*n* = 719	
Age, years	68.9 (68.3, 69.5)	69.0 (68.4, 69.6)	68.6 (67.5, 69.7)	0.806
Systolic BP, mmHg	140.7 (139.8, 141.5)	141.5 (140.8, 142.4)	141.1 (139.6, 142.6)	0.373
Diastolic BP, mmHg	78.0 (77.6, 78.4)	78.5 (78.0, 78.9)	78.4 (77.6, 79.2)	0.258
Hypertension, *n* (%)	1373 (51%)	1411 (53%)	373 (50%)	0.302
Adults 18 to 70 years	*n* = 1124	*n* = 1138	*n* = 313	
Age, years	56.3 (55.4, 57.1)	56.4 (55.6, 57.3)	55.8 (54.2, 57.5)	0.835
Systolic BP, mmHg	135.0 (133.9, 136.0)^a^	136.1 (135.0, 137.2)^ab^	137.6 (135.5, 139.6)^b^	0.026
Diastolic BP, mmHg	79.4 (78.9, 80.0)^a^	80.0 (79.4, 80.5)^ab^	81.4 (80.3, 82.5)^b^	0.013
Hypertension, *n* (%)	464 (40)	514 (44)	149 (46)	0.072

CC (wild type), CT (heterozygous), TT (homozygous variant), genotypes for the *MTHFR* 677C→T polymorphism

Data are expressed as mean (95% CI) for age, as adjusted mean (95% CI) for blood pressure, and *n* (%) for hypertension

*BP* blood pressure

^a^Hypertension defined as systolic BP of ≥ 140 and/or a diastolic BP of ≥ 90 mmHg [1]

^b^Differences in blood pressure between genotype groups were assessed by one-way ANCOVA with adjustment for age, sex, BMI, alcohol, total cholesterol, antihypertensive drugs use and study cohort following log-transformation of data for normalisation purposes, as appropriate. Different superscript letters (i.e. a, b) within a row indicate significant differences by Bonferroni post hoc test, whilst the same letter (i.e. a, a) indicates no significant differences. Categorical variables were assessed using chi-square analysis

**Table 4:** Antihypertensive drug use in treated participants up to 70 years

	*MTHFR* genotype		
	CC (*n* = 536)	CT (*n* = 590)	TT (*n =* 154)
Drug class			
ARB	149 (28)	167 (28)	53 (34)
ACE	185 (35)	221 (37)	52 (34)
CCB	188 (35)	207 (35)	61 (40)
Diuretic	224 (42)	260 (44)	59 (39)
β-Blocker	180 (34)	187 (32)	54 (35)
α-Blocker	38 (7)	35 (6)	7 (5)
Central alpha antagonist	3 (1)	6 (1)	2 (1)
Drug combination			
1 medication	230 (43)	257 (44)	64 (41)
2 medications	200 (37)	205 (35)	52 (34)
≥ 3 medications	106 (20)	128 (22)	38 (25)

Values are *n* (%)

CC (wild type), CT (heterozygous), TT (homozygous variant), genotypes for the *MTHFR* 677C→T polymorphism

*ARB* angiotensin II receptor blockers, *ACE* angiotensin-converting enzyme, *CCB* calcium-channel blockers

In younger and middle-aged adults (18–65 years), significantly lower treated and controlled rates (defined as taking antihypertensive drugs and a recorded BP within the target range, i.e. systolic BP of < 140 and diastolic BP < 90 mmHg) were observed in the TT genotype (30%; *n* = 24) compared to CT (37%; *n* = 114) or CC (45%; *n* = 120) genotypes (*P* < 0.027); not shown.

### *MTHFR* genotype and riboflavin status in relation to hypertension

**Fig. 3:**
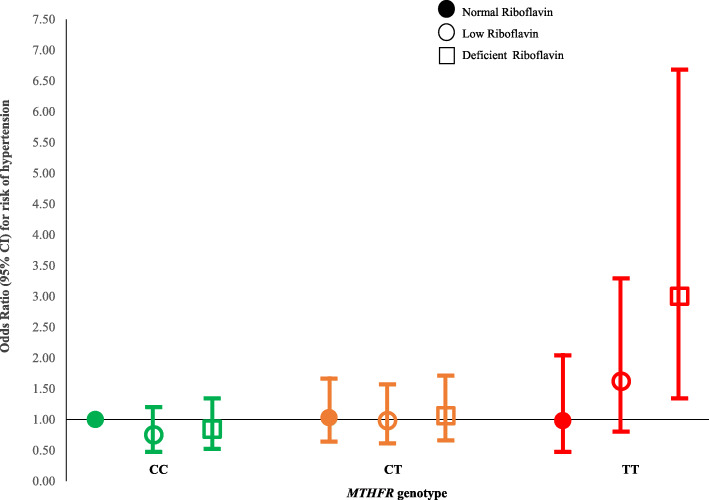
Influence of riboflavin status on the risk of hypertension by *MTHFR* genotype. Values are odds ratios (95% confidence intervals) for risk of hypertension for CC (left panel, green), CT (middle panel, amber) or TT (right panel, red) genotypes for the *MTHFR* 677C→T polymorphism. Data analysed by multinomial regression adjusted for relevant covariates: age, sex, BMI, alcohol, antihypertensive drug use, total cholesterol, creatinine, smoking, study cohort, plasma homocysteine, red blood cell folate. Compared to the reference category (CC genotype combined with normal riboflavin status), values for the TT genotype combined with deficient riboflavin status are OR 3.00 (95% CI, 1.34–6.68; *P* = 0.007); or with low riboflavin status: OR 1.62 (0.80–3.29; *P* = 0.179); or with normal riboflavin status: OR 0.98 (0.47–2.04; *P* = 0.957). Riboflavin status determined by the functional biomarker, erythrocyte glutathione reductase activation coefficient (EGRac); participants categorised as having normal (EGRac ≤ 1.26; filled circles), low (EGRac > 1.26 to < 1.40; open circles) or deficient (EGRac ≥ 1.40; open squares) riboflavin status

The influence of riboflavin status in modifying the genetic risk of hypertension was then examined (Fig. 3). Based on functional status and response to low-dose riboflavin from previous reports [17], participants were categorised as having normal (EGRac ≤ 1.26), low (EGRac 1.26–1.40) or deficient (EGRac ≥ 1.40) riboflavin. Low or deficient riboflavin status (observed in 30.2% and 30.0%, respectively) exacerbated the risk of hypertension associated with this polymorphism, with a 3-fold increased risk (OR 3.00) for the TT genotype in combination with deficient riboflavin status (95% CI, 1.34–6.68; *P* = 0.007) relative to the CC genotype combined with normal riboflavin status as the reference category (Fig. 3). Among participants with the TT genotype, better riboflavin status was associated with a reduced risk (OR 1.62 (95% CI, 0.80–3.29; *P* = 0.179)) and normal riboflavin status with no excess genetic risk of hypertension. In contrast, deficient versus low versus normal riboflavin status did not alter the risk of hypertension among adults with CC or CT genotypes.

## Discussion

Our study shows that from young adulthood to 70 years, the *MTHFR* 677TT genotype predisposes an individual to a systolic BP typical of an adult several years older without this genetic variant. Although this polymorphism was previously linked with BP, this is the first study to examine the genetic risk of hypertension throughout adulthood and to identify the potential for riboflavin to modify the phenotype in affected adults at a younger age and before the onset of hypertension. The observed effect of *MTHFR* and its modulation by riboflavin in relation to hypertension risk were found to be independent of homocysteine, the typically reported phenotype linking this polymorphism with CVD.

We observed a pattern in the current study (irrespective of *MTHFR* genotype), whereby systolic BP increased into older age whereas diastolic BP increased until about 60 years and then declined, as previously reported [21, 22]. The results however showed that adults with the variant *MTHFR* 677TT genotype have higher systolic and diastolic BP compared to others of the same age with CC or CT genotypes. The BP phenotype was not evident above 70 years, presumably as a result of the confounding effect of other age-related determinants of BP. The reason we focussed on the period up to 70 years is because this is a time during which the relationship of BP with disease is most pronounced, with a reported doubling in the risk of CVD for each 20 mmHg rise in systolic BP [4]. The *MTHFR* 677TT genotype was associated with a 42% increased risk of hypertension in adults up to 70 years, after adjustment for antihypertensive drug use and other significant factors, namely, age, male sex, BMI, alcohol and blood cholesterol, whereas plasma homocysteine was not independently associated with hypertension risk. The extent of excess hypertension owing to this polymorphism is in good agreement with previous estimates from clinical studies, with reported odds ratios in meta-analyses ranging 1.36 (95% CI, 1.20–1.53) to 1.87 (1.31 to 2.68), for worldwide and Chinese populations, respectively [8, 10]. Our findings however show that from young adulthood, this polymorphism contributes to higher BP, suggesting that affected adults could potentially develop hypertension at an earlier age than those without this genetic risk.

Of particular relevance to cardiovascular medicine is the finding that response to routine BP treatment appears to be suboptimal in adults with the *MTHFR* 677TT genotype. Overall, 49% of participants 18–70 years in this study were under current treatment with antihypertensive drugs, a rate of treatment similar to that reported for adults 20–80 years in England (51%) and considerably less than in adults 20–80 years in the US (74%) or Canada (80%) [21]. In the current study, in line with our previous observations [17], BP control was poorer in the TT genotype, with only 30% of treated adults with the TT compared to 37% in CT and 45% in CC genotypes, achieving BP control. Similarly, reported BP control rates for all treated adults are 37% in England [23] and higher in North America, at 54% in the US [5] and 65% in Canada [23]. Irrespective of prevailing rates of treatment or BP control, however, our findings suggest that within a given population, adults with the TT genotype compared to others without this gene variant will be less likely to achieve target BP with routine treatment, but neither the patient nor the physician will be aware of this. The economic implications of suboptimal BP control are considerable, with the direct costs of hypertension estimated in 2009 at $370 billion annually, representing 10% of healthcare expenditures worldwide [24].

Uniquely, this study enabled the genetic risk of hypertension owing to this polymorphism to be considered in relation to riboflavin (the MTHFR cofactor). Unlike other B vitamins (e.g. folate and vitamin B12), riboflavin biomarkers are rarely measured in human studies and no previous cohort study to have investigated this polymorphism has considered riboflavin [25]. We estimated a 3-fold increased risk of hypertension when the variant TT genotype occurred in combination with deficient riboflavin status (relative to the CC genotype and normal riboflavin status), whereas better riboflavin status was associated with reducing the excess hypertension risk, and normal riboflavin status with no genetic risk. In contrast, among adults with CT or CC genotypes, riboflavin status did not influence the risk of hypertension, evidence that riboflavin has a genotype-specific role in BP. The finding that riboflavin has the potential to modify BP in adults affected by this polymorphism is entirely consistent with our earlier studies in hypertensive patients, which showed a lowering of systolic BP by 6 to 13 mmHg in response to riboflavin supplementation specifically in the TT genotype [15–17], resulting in a marked increase in blood-pressure control from 32 to 57% (pre versus post riboflavin intervention for 16 weeks), despite no change in antihypertensive treatment over the intervention period [17]. Here we show the potential of riboflavin to modify BP in genetically at-risk adults at an earlier age and the data suggest that the onset of hypertension could be delayed through intervention with riboflavin. Ideally, such intervention would occur prior to commencing antihypertensive treatment and along with lifestyle interventions as per ESC/ESH guidelines for hypertension management [1], especially considering that riboflavin is safe with no known adverse effects even at doses of 100-fold higher than typical dietary intakes [26]. Alternatively, riboflavin could be co-administered with an antihypertensive drug as a novel combination therapy targeted at patients with this genetic risk factor. The potential to prevent or treat hypertension in sub-populations worldwide could be considerable, given that this genotype affects 10% of people globally, ranging 4–26% in Europeans (increasing north to south), 20% in Northern China, to as high as 32% in Mexico [11].

The impact of this polymorphism on BP throughout adulthood and the potential modifying effect of riboflavin are important findings, considering that this polymorphism is linked with an increased risk of stroke [12–14], and recent evidence shows that living longer in better cardiovascular health during mid-life is associated with lower risk of disease and mortality later in life [27]. Control of BP is highly effective in reducing cardiovascular mortality [5, 23, 24], with each 2 mmHg lower systolic BP associated with a 10% lower risk of stroke [4]. Furthermore, powerful evidence, from the SPRINT trial testing the effects of intensive versus standard blood-pressure control [28] and from meta-analyses of large-scale BP lowering trials [29], highlights significant benefits for cardiovascular risk especially among middle-aged adults [30] of BP-lowering to values below hypertension cut-points. Because of concerns that intensive treatment of BP could also pose certain risks [31], however, there have been calls for newer approaches, including novel combination therapies and non-pharmacological solutions [32]. Our results indicate that the most effective timeframe to target adults with this genetic variant will be up to 70 years, via supplementation with riboflavin to potentially offer an effective low-cost strategy to lower BP. Of note, sub-optimal riboflavin status may be more widespread than is generally recognised, but is largely undocumented as riboflavin biomarkers are rarely measured in human studies [25]. The UK is one of the very few countries worldwide to include a riboflavin biomarker in its population-wide diet and nutrition survey, and recent data shows that over 50% of healthy British adults have low or deficient riboflavin status [33], in close agreement with the current results in Irish adults.

The biological mechanism explaining MTHFR-BP relationship shown here is unknown, but likely involves the potent vasodilator nitric oxide (NO) [34]. Vascular tissue concentrations of 5-methyltetrahydrofolate (the product of the MTHFR reaction) were associated with NO bioavailability and improved endothelial function in patients undergoing coronary artery bypass graft surgery and were found to be lower in those patients with the TT genotype [35, 36]. The current results, considered with our earlier trials [15–17], indicate that the biologic perturbation leading to higher BP in the TT genotype is modifiable with riboflavin. Molecular studies show that the decreased enzyme activity in the TT genotype is owing to loss of the riboflavin (FAD) cofactor from the active site [37], but riboflavin intervention can restore MTHFR activity in vivo [38]*.* Restoring MTHFR in vascular tissue could in turn lower BP specifically in individuals with the TT genotype. Mechanistic studies are required, but at this time, the evidence does not support a direct role for homocysteine in BP. Although elevated homocysteine is the characteristic phenotype linked with this polymorphism (and is responsive to riboflavin in the TT genotype [38]), intervention trials to lower homocysteine have shown no corresponding BP response [39], indicating that homocysteine is not causatively related to hypertension. The current results suggest that this polymorphism is linked with CVD via BP independently of homocysteine, and given its importance for clinical outcomes, BP may be the much more relevant target to prevent CVD in those affected by the variant genotype.

A strength of this study is its large sample of adults 18 to 90 years stratified for the relevant polymorphism using data from two cohorts sampled under a common project initiative, from participating centres in Northern Ireland (UK) and the Republic of Ireland (representing two distinct health systems), using standardised methodologies and centralised laboratory analysis to investigate outcomes that were formulated before data collection. Furthermore, the current analysis was based on an a priori hypothesis (linking this polymorphism and riboflavin with BP) whereas other studies of genetic risk factors in relation to disease risk factors are typically opportunistic studies using available data. Thus uniquely, our study provides biomarker data for riboflavin, rarely measured in nutritional studies, and used here to enable the impact of riboflavin on the MTHFR-BP relationship from young adulthood to be demonstrated. The major limitation of this study is its cross-sectional (rather than a longitudinal) design; nonetheless, the study findings in relation to the genotype-specific effect of riboflavin are reinforced by our earlier trials [15–17] showing significant BP-lowering in response to intervention with riboflavin in CVD patients identified with the relevant genotype.

## Conclusion

The variant *MTHFR* 677TT genotype is associated with higher BP independently of homocysteine and predisposes adults to an increased risk of hypertension and poorer BP control with antihypertensive treatment, whilst better riboflavin status is associated with a reduced genetic risk. Supplemental riboflavin could therefore offer a stratified approach to delay the onset of hypertension and/or improve blood-pressure control in adults with the TT genotype, representing 10% of people globally and higher in some populations. Such an approach aligns with international strategies of personalising treatments to improve cardiovascular health, but the findings require confirmation in randomised trials in non-hypertensive adults.

## Supplementary information

**Additional file 1: Table S1** General study characteristics by NANS and TUDA cohorts. **Table S2** Blood pressure and use of antihypertensive drugs in adults 18–70 years by *MTHFR* genotype.

## Data Availability

Data from this study are held in full compliance with Ulster University’s Research Governance and Ethics Policy for Human Research (2018) https://internal.ulster.ac.uk/research/office/rofficeeg.php, which in turn is fully aligned with the UK’s Data Protection Act 2018. The data underlying the results presented in the study are available from Mr. Nick Curry, Head of Research Governance at Ulster University at n.curry@ulster.ac.uk.

## Abbreviations

BP: Blood pressure
CVD: Cardiovascular disease
EGRac: Erythrocyte glutathione activation coefficient
FAD: Flavin adenine dinucleotide
GWAS: Genome-wide association studies
MTHFR: Methylenetetrahydrofolate reductase
NANS: National Adult Nutrition Survey
SNPs: Single nucleotide polymorphisms
TUDA: Trinity-Ulster Department of Agriculture
