# Supplementary material for: Impact of the common MTHFR 677C→T polymorphism on blood pressure in adulthood and role of riboflavin in modifying the genetic risk of hypertension: evidence from the JINGO project
Source: BMC Med. 2020 Nov 11;18:318. doi: 10.1186/s12916-020-01780-x (PMC7656675; doi:10.1186/s12916-020-01780-x)
Supplement: Supplementary file 1 — Additional file 1: Table S1 General study characteristics by NANS and TUDA cohorts. Table S2 Blood pressure and use of antihypertensive drugs in adults 18–70 years by MTHFR genotype. [file 12916_2020_1780_MOESM1_ESM.docx]

**Table S1** General study characteristics by NANS and TUDA cohorts

|  | NANS cohort^a^  (*n* = 1021) | TUDA cohort^a^  (*n =* 5055) |
| --- | --- | --- |
| General characteristics |  |  |
| Age, years | 43.4 (16.5) | 74.1 (8.3) |
| Sex, male | 504 (49.4%) | 1656 (32.8%) |
| Waist, cm | 91.2 (13.5) | 95.2 (14.1) |
| Height, cm | 169.4 (9.7) | 161.4 (9.7) |
| Weight, kg | 77.6 (15.5) | 73.0 (16.9) |
| Body mass index, kg/m^2^ | 26.9 (4.5) | 27.9 (5.4) |
| Current smokers | 19.5 | 11.9 |
| Alcohol Intake, units/week | 10.6 (11.4) | 7.9 (12.5) |
| Serum triglycerides, mmol/l | 1.31 (0.78) | 1.58 (0.86) |
| Serum total cholesterol, mmol/l | 4.96 (1.00) | 4.63 (1.05) |
| Serum HDL, mmol/l | 1.57 (0.44) | 1.48 (0.48) |
| Calculated LDL, mmol/l | 2.80 (0.86) | 2.44 (0.88) |
| Systolic blood pressure, mmHg | 124.7 (17.8) | 144.4 (21.1) |
| Diastolic blood pressure, mmHg | 78.1 (10.9) | 78.2 (11.2) |
| Serum Creatinine, µmol/l | 89.6 (14.4) | 85.4 (28.8) |
| B vitamin biomarkers and supplement use |  |  |
| B vitamin supplement use, %**^a^** | 15 | 14 |
| Red blood cell folate, nmol/l | 970 (443) | 1100 (625) |
| Serum vitamin B12, pmol/l | 326 (186) | 289 (208) |
| Riboflavin status, EGRac^b^ | 1.37 (0.18) | 1.34 (0.21) |
| Plasma homocysteine, µmol/l | 12.4 (3.9) | 14.9 (5.8) |
| *MTHFR* genotype frequency, %^c^ |  |  |
| CC | 44.6 | 44.0 |
| CT | 43.1 | 43.9 |
| TT | 12.3 | 12.1 |

Data expressed as mean (SD) or *n* (%).

Abbreviations: NANS, National Adult Nutrition Survey of Ireland; TUDA, Trinity-Ulster and Department of Agriculture study

^a^Including riboflavin

^b^Biomarker status of riboflavin determined by the functional assay, erythrocyte glutathione reductase activation coefficient (EGRac); higher values indicate lower riboflavin status.

^c^CC (wild type), CT (heterozygous), TT (homozygous variant) genotypes for the *MTHFR* 677C→T polymorphism.

**Table S2** Blood pressure and use of antihypertensive drugs in adults 18-70 years by *MTHFR* genotype

|  | *MTHFR* genotype | | |  |
| --- | --- | --- | --- | --- |
|  | CC | CT | TT | *p* value^1^ |
| Total cohort | *n* = 1124 | *n* = 1138 | *n* = 313 |  |
| Age, years | 56.3 (55.4, 57.1) | 56.4 (55.6, 57.3) | 55.9 (54.3, 57.5) | 0.835 |
| Systolic blood pressure, mmHg | 135.0 (133.9, 136.0)^a^ | 136.1 (135.0, 137.2)^ab^ | 137.6 (135.5, 139.9)^b^ | 0.026 |
| Diastolic blood pressure, mmHg | 79.4 (78.9, 80.5)^a^ | 80.0 (79.4, 80.5)^ab^ | 81.4 (80.3, 82.5)^b^ | 0.013 |
| Hypertension, n (%) | 464 (40%) | 514 (44%) | 149 (46%) | 0.072 |
| Not taking antihypertensive drugs | *n* = 595 | *n* = 560 | *n* = 163 |  |
| Age, years | 49.0 (47.8, 50.3) | 48.1 (46.8, 49.5) | 47.9 (45.4, 50.3) | 0.451 |
| Systolic blood pressure, mmHg | 126.8 (125.5, 128.1)^a^ | 127.5 (126.2, 128.8)^ab^ | 130.4 (128.0, 132.8)^b^ | 0.017 |
| Diastolic blood pressure, mmHg | 77.8 (77.0, 78.6)^a^ | 78.5 (77.7, 79.4) ^ab^ | 80.2 (78.7, 81.7)^b^ | 0.019 |
| Hypertension, n (%) | 155 (25%) | 138 (24%) | 52 (31%) | 0.179 |
| Taking antihypertensive drugs | *n* = 529 | *n* = 578 | *n* = 150 |  |
| Age, years | 64.6 (64.2, 64.9) | 64.5 (64.2, 64.8) | 64.5 (63.8, 65.1) | 0.961 |
| Systolic blood pressure, mmHg | 143.6 (142.0, 145.2) | 145.0 (143.5, 146.6) | 145.5 (142.4, 148.5) | 0.339 |
| Diastolic blood pressure, mmHg | 81.2 (80.3, 82.0) | 81.5 (80.7, 82.3) | 82.7 (81.1, 84.3) | 0.395 |
| Hypertension, n (%) | 309 (58%) | 376 (64%) | 97 (63%) | 0.099 |

Abbreviations: CC (wild type), CT (heterozygous), TT (homozygous variant), genotypes for the *MTHFR* 677C→T polymorphism.

Data are expressed as adjusted mean (95% CI) or *n* (%).

^1^Differences in blood pressure between genotype groups were assessed by one-way ANCOVA with adjustment for age, sex, BMI, alcohol consumption, serum total cholesterol and study cohort following log-transformation of data for normalization purposes, as appropriate. Different superscript letters (i.e. a, b) within a row indicate significant differences by Bonferroni post-hoc test, whilst the same letter (i.e. a, a) indicates no significant differences. Categorical variables were assessed using chi-square analysis.
